# Cooking Well with Diabetes: A Healthy Cooking School for Diabetes Prevention and Management

**DOI:** 10.3390/nu16152543

**Published:** 2024-08-03

**Authors:** Sumathi Venkatesh, David O. Leal, Amy Valdez, Paula I. Butler, Odessa E. Keenan, Elaine Montemayor-Gonzalez

**Affiliations:** 1 Department of Nutrition, Texas A&M AgriLife Extension Service, College Station, TX 77840, USA; 2 Healthy South Texas, Texas A&M AgriLife Extension Service, College Station, TX 77845, USA; 3 Family and Community Health, Texas A&M AgriLife Extension Service, Dallas, TX 75252, USA

**Keywords:** type 2 diabetes, nutrition education, healthy cooking

## Abstract

One in ten Americans suffers from type 2 diabetes, which, if not managed well, can result in severe complications, disability, and premature death. Diabetes education classes can play a pivotal role in providing practical education on diabetes and self-care behaviors, with a particular emphasis on dietary management, which is often regarded as the most demanding diabetes self-care behavior. The Texas A&M AgriLife Extension Service developed Cooking Well with Diabetes (CWWD), a four-week interactive diabetes education series, with each week consisting of a lecture on healthy eating coupled with cooking lessons featuring diabetes-friendly recipes. The current study aimed to examine the effectiveness of CWWD in improving the frequency of healthy food preparation and consumption of program participants. Secondary data from 2017 to 2023 was analyzed involving 1574 adults from 59 predominantly rural Texas counties. Data from self-reported pre and post evaluations showed improvements in healthy food preparation and consumption behaviors. The curriculum enabled Extension Educators to introduce healthful dietary behaviors to a diverse group of clients. The curriculum can be adapted by Extension Educators in other states reaching a broader audience. The findings will inform future research aimed at planning and implementing successful diabetes education programs.

## 1. Introduction

Type 2 diabetes affects 11.3% of Americans, with another 38% suffering from prediabetes [1]. The rising prevalence of diabetes and obesity, high uninsured rates, and limited access to health care all contribute to a less-than-desirable quality of life for a significant proportion of Texans. Untreated diabetes or poor blood glucose control can lead to several complications, such as heart disease, stroke, kidney disease, blindness, increased time away from work, and reduced quality of life [2,3,4]. Self-care behaviors, along with medical monitoring of blood glucose, may help reduce healthcare costs and medical complications associated with this condition [4]. The American Association of Diabetes Care and Education Specialists (ADCES) recommends healthy eating, regular exercise, home blood glucose monitoring, medication adherence, healthy coping, problem solving, and risk reduction behaviors as key diabetes self-management practices [5].

For many, adhering to a healthy eating pattern is a challenging aspect of diabetes self-management [6,7,8,9,10]. Research shows that the cost and accessibility of nutritious foods, cultural food practices, and difficulties in adjusting to a diabetes-friendly diet are major barriers to healthy eating [6,7,8,9,10]. Without essential cooking skills and knowledge about recommended foods and portion sizes, meal preparation can become quite challenging [6,7,8,9,10]. Social barriers, such as a lack of social support or the need to prepare separate meals for family members with different dietary needs, can pose additional challenges [6,9,10]. Addressing these barriers by educating individuals with type 2 diabetes is crucial for improving their self-efficacy in diabetes self-management [6,8,10,11]. Therefore, a hands-on approach to diabetes education could help overcome these barriers to cooking and consuming healthy foods.

Diabetes education focusing on healthy cooking and carbohydrate monitoring can inform individuals and their support networks about dietary management for blood glucose control. The Cooperative Extension System (CES) has a far-reaching presence in nearly 3000 counties across the United States, making it an ideal platform for hosting educational programs that address critical issues like diabetes [12]. Therefore, the Texas A&M AgriLife Extension Service developed “Cooking Well with Diabetes” (CWWD), a community-based, hands-on diabetes cooking education program for adults in English and Spanish, to promote behavior change and improve diabetes-related health outcomes through healthy eating and lifestyle changes. The program provides education on carbohydrate containing foods, their impact on blood glucose levels, and healthy food preparation methods to help individuals with diabetes and their support networks. The objective of this study was to examine the effectiveness of CWWD in improving the frequency of healthy food preparation and consumption of program participants.

## 2. Materials and Methods

The Texas A&M University Institutional Review Board approved the study to analyze and publish secondary data collected from the CWWD program between 2017 and 2023. The Texas A&M AgriLife Extension Service developed the CWWD program series in 2004 based on the recommendations from the American Diabetes Association, the Academy of Nutrition and Dietetics, and Current Trends in Medical Nutrition Therapy. In 2016, a team of AgriLife Extension personnel with expertise in nutrition research and chronic disease program development and outreach (the curriculum development team) redesigned the program series, incorporating updated information, new recipes, and Spanish translations. Further updates were made in 2020, including a refreshed look, updated content and handouts, new marketing materials, and translation updates.

CWWD consists of four weekly lessons, each followed by a hands-on cooking session. The lessons focus on understanding the carbohydrate content in food, reducing sodium and fat, increasing dietary fiber, and practicing portion control. Participants were educated on carbohydrate counting and the diabetes plate method as effective tools for monitoring their carbohydrate intake. Various educational resources were provided to help participants apply these concepts beyond the classroom. Table 1 illustrates examples of carbohydrate choices in various foods. A carbohydrate choice is defined as a serving of food containing approximately 15 g of carbohydrates.

Each lesson included six diabetes-friendly recipes developed with a variety of ingredients and healthy preparation methods. The recipes met the USDA dietary guidelines for sodium, saturated fat, and added sugars. The recipes were created using ESHA Research Food Processor ™ Nutrition Analysis Software version 11.11.32. Repeated nutritional analysis was conducted, and the recipes were tested for taste, acceptability, and nutritional quality. Some examples of recipes used for the demonstrations were baked parmesan catfish (each fillet serving provides 230 calories and 16 g of carbohydrates), hearty two-bean minestrone (a 400 g serving provides 210 calories and 32 g of carbohydrates), and pumpkin pie parfait (each serving provides 96 calories and 17 g of carbohydrates). The recipes were adapted from another Texas A&M AgriLife Extension nutrition program, Dinner Tonight Healthy Cooking School available at https://dinnertonight.tamu.edu (accessed on 8 February 2024).

The curriculum development team regularly conducted training sessions for Extension Educators on program content and lesson delivery. Over the past five years, four regional training sessions were held both in-person and via virtual platforms, with good attendance from Extension Educators across Texas. The training covered program materials, marketing materials, program delivery, and provided an in-depth overview of the lessons and recipes. The curriculum development team was available for guidance at any time.

Extension Educators regularly met with their county health coalitions and program committee members to review the county diabetes risk profile and assess the need for a diabetes education program in their respective counties. The committee determined the target audience, implementation sites, and chose appropriate delivery methods for their county. Extension Educators marketed the program to their clients through email communication and by posting program flyers on social media sites, AgriLife Extension websites, and partner websites. Interested participants contacted the Extension Educators using the contact information provided in the flyer. Members of the Texas Extension Education Association, AgriLife Extension Master Wellness volunteers, health coalition members, and Family and Community Health program area committee members assisted the Extension Educators with instruction and recipe demonstrations. The Master Wellness program, an AgriLife Extension volunteer initiative, provides health and nutrition training and certification to adults. Once certified, Master Wellness volunteers assist Extension Educators with education and outreach.

**Figure 1:**
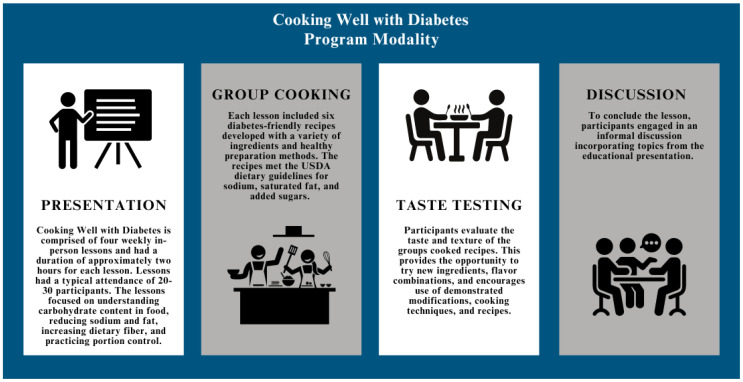
Program Modality of the Cooking Well with Diabetes Program.

The program modality is presented in Figure 1. Weekly in-person classes were held, each lasting approximately two hours per session. Sessions typically had an attendance of 20–30 participants. The class format included time for PowerPoint presentation, group recipe preparation, tasting, and informal discussion. The program was open to individuals eighteen and older, including those with type 2 diabetes, their support networks, those at risk of developing type 2 diabetes, and those interested in preventing type 2 diabetes. The program cost varied among counties based on funding availability, generally ranging from $20 to $40 for the entire series. Extension Educators determined program cost by calculating program resources and recipe ingredient costs. Fee waivers or scholarships were provided by local counties for individuals with limited resources.

In response to the 2020 COVID-19 pandemic, the curriculum development team obtained feedback from a representative sample of Extension Educators to determine additional materials needed for virtual programming and online course publication. The curriculum development team updated the curriculum with new handouts, marketing materials (including flyers, news releases, and social media marketing), and program planning and implementation guides for virtual classes. All materials and resources were designed to be relevant for diverse audience groups.

After receiving training in virtual program delivery, Extension Educators conducted classes through virtual platforms such as Microsoft Teams version 24071128817, Zoom version 6.0.11, and private Facebook groups. Extension Educators used live streams or recordings of lessons and recipe demonstrations, allowing participants to engage with the educators. On Facebook groups, educators posted reminders, infographics, and interactive content daily. Additionally, the curriculum development team pilot tested and published the CWWD program as an asynchronous online course on the Texas A&M AgriLife Extension digital learning platform (https://agrilifelearn.tamu.edu (accessed on 8 February 2024)). Extension Educators marketed the program to their clients through email communication and by posting on social media sites.

Program outcomes were captured using pre and post surveys completed during the first and last educational sessions, respectively. Evaluations were administered either via the Qualtrics survey platform or using scannable paper-based surveys. The evaluation measures included participants’ demographic information, diabetes diagnosis, perception of their general health on a five-point scale (ranging from excellent to poor), and time spent in moderate or vigorous intensity activities during the past week. Participants indicated their frequency of healthy cooking behaviors on a five-point scale, ranging from always to never. Daily consumption of fruits, vegetables, sodas, and sugar sweetened beverages was measured using a seven-point scale, ranging from never to four or more times a day. Virtual and online participants completed the same pre and post surveys as those used for in-person programming.

The Qualtrics surveys were managed and analyzed by the Office of Data and Accountability (ODA) at Texas A&M AgriLife Extension Service. Extension Educators mailed paper-based evaluations to ODA, where they were scanned and analyzed to produce outcome reports. Pre- and post-data were analyzed to determine the percentage of participants reporting improvements in healthy food behaviors compared to their baseline information. Chi-square tests on matched surveys examined the percentage of participants reporting improvements in knowledge, food choices, and healthy food behaviors after completing the four-week series compared to their baseline information. A *p*-value < 0.05 was considered statistically significant.

## 3. Results

### 3.1. Participant Characteristics

Participant characteristics are reported in Table 2. CWWD was implemented in 59 counties across Texas, predominantly rural with only four urban areas. Most program participants were from the east, southeast, and southern regions of Texas (92.5%). From 2017 to 2023, the program reached 1574 participants through face-to-face, virtual, and online classes. Of these, 1339 (85.1%) completed English evaluations, and 131 (8.3%) completed Spanish forms. Most program evaluations were collected via scannable surveys (*n* = 1470; 93.4%), with a few collected through Qualtrics surveys (*n* = 104; 6.6%). The age range of participants was 18 to 98 years, and the majority were female (*n* = 1195; 75.9%). Forty-four percent (*n* = 706) identified as Hispanic, 34.9% (*n* = 550) as non-Hispanic white, 14.4% (*n* = 227) as African American, 1.2% (*n* = 19) as American Indian or Alaska Native, and 2.3% (*n* = 35) as other (Asian, multiracial, Native Hawaiian or Pacific Islander, or other). Of the total sample, 48% (*n* = 755) reported having type 2 diabetes, 12.7% (*n* = 200) had participated in other diabetes cooking schools, 32.8% (*n* = 517) prepared meals for someone with diabetes, and 85.3% (*n* = 1342) were the primary person responsible for food shopping in the household.

### 3.2. General Health, Physical Activity, and Nutrition

Compared to baseline data, program outcomes improved at the time of follow-up. Participants’ perceptions of their general health, engagement in moderate and vigorous intensity physical activity, and consumption of fruits, vegetables, regular soda, and sugar sweetened beverages (such as fruit punch, fruit drinks, sweet tea, or sports drinks) significantly improved after completing the program series (Table 3). Likewise, the proportion of participants consuming fruit (pre 34.8% vs. post 49.0%) and vegetables (pre 47.0 vs. post 62.2%) two or more times a day, as well as those who reduced their consumption of regular sodas and sweetened beverages, improved at follow-up.

### 3.3. Healthy Cooking Practices

Program participants reported always or mostly engaging in healthy cooking practices (see Table 4) such as not adding salt at the table (pre 17.0% vs. post 15.6%), replacing salt with herbs and spices (pre 33.4% vs. post 46.6%), replacing solid fat with vegetable oil (pre 68.4% vs. post 80.6%), modifying recipes to reduce sugar or fat (pre 32.6% vs. post 43.5%), increasing dietary fiber (pre 21.5% vs. post 33.1%), using healthy cooking methods such as broiling, baking, or grilling instead of frying (pre 55.2% vs. post 72.4%), adding extra vegetables to mixed dishes such as casseroles and soups (pre 15.6% vs. post 32.2%), planning their meals using the MyPlate recommendations (pre 15.6% vs. post 32.2%), consulting nutrition facts labels (pre 34.6% vs. post 53.8%), and preparing meals and snacks at home (pre 63.2% vs. post 72.9%).

## 4. Discussion

The study aimed to examine the effectiveness of CWWD, a hands-on community-based diabetes education program, in improving the frequency of healthy food preparation and consumption among program participants. In the 59 counties where CWWD was implemented, most program participants showed an improvement in both the frequency of healthy food preparation and consumption.

Dietary intake plays a significant role in blood glucose management [13,14]. Carbohydrate intake is particularly important in diabetes management, as it is the main source of glucose and the primary factor in insulin secretion [15]. Carbohydrate counting and the diabetes plate method are two meal planning approaches used in diabetes self-management education, both of which have shown promising results [16]. The CWWD curriculum incorporated these two dietary strategies to educate program participants on carbohydrate control and identifying healthy carbohydrate sources.

Nutrition education is a key component in improving dietary intake and creating healthy lifestyle changes to optimize glycemic control and, ultimately, prevent or manage diabetes [13,14,17,18,19,20]. Several research studies have demonstrated that nutrition education can enhance nutritional and health outcomes beneficial for diabetes management [21]. Specifically, a hands-on approach that combines nutrition education with a cooking intervention has been shown to increase fruit and vegetable consumption and reduce intake of calories, carbohydrates, total fat, saturated fat, cholesterol, and sodium [22,23].

Cooking programs with hands-on participation have not only enhanced food intake and dietary behaviors but have also demonstrated improvements in clinical outcomes. A retrospective chart review by Byrne et al. showed that a diabetes self-management cooking program, which focused on affordable recipes and food choices, improved hemoglobin A1c levels. Greater improvements in glycemic control were correlated with increased attendance at the classes [24]. Consistent with our findings, a study by Williams et al. conducted a six-week diabetes education program combined with a cooking intervention for 48 participants with either type 1 or type 2 diabetes. The authors assessed participants’ diet quality by calculating Healthy Eating Index scores from their past 30-day dietary intake, collected using the Diet History Questionnaire. The study documented improvements in diet quality, with higher vegetable intake and lower consumption of refined grains, added sugars, and calories post intervention compared to baseline [22]. This approach of integrating nutrition knowledge with practical training can play a powerful role in supporting healthier nutrient intakes, diet-related self-care, and quality of life among participants with type 2 diabetes [22].

CWWD incorporated visual learning by teaching program participants using illustrations that included Choose MyPlate, the diabetes plate method, carbohydrate counting, and reading nutrition facts labels, all of which have been shown to facilitate glycemic control [25]. Studies conducted among individuals with type 2 diabetes have clearly indicated that carbohydrate counting and utilizing a modified plate method are effective in improving hemoglobin A1c levels [16,26]. Furthermore, CWWD utilizes recipe collections from another Texas A&M AgriLife Extension nutrition program, Dinner Tonight Healthy Cooking School, to support healthy eating by incorporating nutrient-rich foods and limiting saturated fats, sodium, and cholesterol. These recipes are easy to prepare and follow the recommendations of the 2020–2025 Dietary Guidelines for Americans.

Diabetes lifestyle programs that focus on meal planning, healthy eating, and active living have shown positive outcomes for participants and fall within the scope of practice for most Extension Educators. Utilizing and expanding evidence-based diabetes education through the CES is a potential way to bridge the gap for communities lacking educational programs. A few diabetes education programs have been implemented using the CES, including an evidence-based curriculum developed by the Centers for Disease Control and Prevention (CDC) [27]. The most widely implemented diabetes self-management education and support (DSMES) program is the CDC’s National Diabetes Prevention Program (NDPP or DPP). However, this program lacks a cooking intervention and has limited adoption among Extension Educators [28]. To address these challenges, CWWD offers a cost-effective curriculum with experiential learning, encompassing dietary management of type 2 diabetes in a comprehensive five-week series.

Integrated nutrition and cooking education implemented by Extension professionals have shown promising results, including increased likelihood of tight glycemic control and improvements in self-efficacy, self-care practices, and healthy lifestyle behaviors [29,30]. For example, the New Mexico Cooperative Extension implemented “Kitchen Creations,” a hands-on cooking program aimed at assessing changes in nutrient intake among individuals with type 2 diabetes from diverse ethnic and socioeconomic backgrounds. Analysis of dietary data collected using a three-day food record showed significant reductions in carbohydrate, saturated fat, dietary cholesterol, and sodium intake following the program. Additionally, the program successfully reduced overall energy intake and the percentage of calories from fat, which supports healthy weight management for individuals with type 2 diabetes [23]. Thus, the CES has the potential to be an ideal platform for hosting diabetes education interventions due to its extensive presence across the United States. Increasing the number of evidence-based diabetes education programs available for Extension Educators and other nutrition/health educators could further promote the adoption of such programs across the United States.

Community-based health education facilitates broad outreach and program participation. The CWWD curriculum supports the American Diabetes Association Standards of Care by providing individuals with diabetes practical tools to promote healthful eating patterns [31]. This includes evidence-based approaches such as the diabetes plate method and carbohydrate counting, which are effective in achieving improved glycemic control [16]. An important strength of our study is the hands-on approach that allowed participants to engage in experiential learning and interact with instructors. Our study included a considerable sample size, enhancing the generalizability of the findings. Our recipes and nutrition education adhered to current dietary recommendations and evidence-based practices.

However, there were a few limitations. We relied on self-reported health and dietary information, which may have led to over- or under-representation of the actual intake and practices. Additionally, a sizable number of participants had attended other diabetes education programs before enrolling in CWWD. This prior knowledge may have influenced our findings, as these participants might have been more inclined to adopt the healthy food and meal preparation behaviors presented in our program. The lack of six-month follow-up data limited our ability to assess long-term behavior changes. We recommend follow-up with health care providers to gain a comprehensive understanding of participants’ health outcomes. The evaluation tools used were intended for internal program evaluation and reporting. More robust evaluation methods, including biomarker assessments, would provide a comprehensive assessment of program effectiveness and offer insights into the clinical impacts of a community-based diabetes education program.

## 5. Conclusions

The CWWD program series promoted positive diabetes self-management practices related to food preparation and consumption. Findings from this study documented improvements in food choices and cooking techniques resulting from participation in the educational series. Achieving adequate glycemic control in individuals with diabetes can substantially lower both direct and indirect health care costs associated with managing diabetes and its related complications. Equipping individuals with diabetes with the skills and techniques for preparing healthy meals can boost their confidence in cooking, leading to improved dietary self-care practices and better long-term blood glucose management. Community classes, such as those offered in a group setting like CWWD, provide an opportunity to connect with others who share similar experiences, fostering support networks that extend beyond the classroom.

Developed in 2004, the curriculum has been updated to align with current diabetes recommendations and trends in medical nutrition therapy. Oversight by Extension Nutrition Specialists ensures that Extension Educators receive proper training to accurately deliver the content. The program offers various delivery options, including traditional synchronous learning with live classroom sessions, asynchronous online courses, and options for Spanish speakers, allowing for flexible participation. Additional research is needed to evaluate the effectiveness of synchronous versus asynchronous adoption of recommended behaviors, as well as the impact of cultural adaptations. There is also an opportunity to examine how partnering agencies and trained Master Wellness volunteers contribute to program delivery and outcomes. Our study demonstrated promising outcomes through various delivery methods. Future research could investigate additional approaches, such as mobile applications offering regular classes, diabetes-friendly recipes, and healthy cooking videos.

A community education program delivered by Extension Educators and trained, Master Wellness volunteers offer broad access, as most counties in the United States have local Cooperative Extension offices. The CES offers informal nutrition education to individuals and communities to enhance their health and well-being. Participants with specific medical nutrition therapy questions are typically advised to consult their healthcare providers. A comprehensive diabetes self-management program could involve collaboration of Extension Educators with health care providers for referrals to cooking education programs. Including biomarker assessments could further broaden the scope of community-based diabetes education programs.

## Figures and Tables

**Table 1:** Carbohydrate food choices covered in lesson 1 of the Cooking Well with Diabetes Program.

Food Category	Food Type	Serving Size	Carbohydrate Choices *
Starch and Whole Grains	Bread	1 slice	1
Starch and Whole Grains	Hot Dog or Hamburger Bun	½ a bun	1
Starch and Whole Grains	English Muffin	½ a muffin	1
Starch and Whole Grains	Bagel (4 ounce)	¼ a bagel	1
Starch and Whole Grains	Pancake (4 inches)	1 pancake	1
Starch and Whole Grains	Tortilla (6 inches)	1 tortilla	1
Starch and Whole Grains	Pasta or Rice	1/3 a cup	1
Starch and Whole Grains	Bran	½ cup	1
Starch and Whole Grains	Cereal, unsweetened	¾ cup	1
Starch and Whole Grains	Cereal, Cooked	1/3 cup	1
Starch and Whole Grains	Crackers, Saltines	6 crackers	1
Starchy Vegetables	Corn	½ cup	1
Starchy Vegetables	Potatoes	½ cup	1
Starchy Vegetables	Yams	½ cup	1
Starchy Vegetables	Sweet Potatoes	½ cup	1
Starchy Vegetables	Winter Squash	1 cup	1
Starchy Vegetables	Baked Beans	1/3 cup	1
Starchy Vegetables	Beans	½ cup	1
Starchy Vegetables	Peas	½ cup	1
Sweets and Desserts	Cake, frosted	2-inch square	2
Sweets and Desserts	Cake, unfrosted	2-inch square	1
Sweets and Desserts	Angel Food Cake	1/12	2
Sweets and Desserts	Glazed Doughnut	3.75 inch	2
Sweets and Desserts	Ice Cream	½ cup	1
Sweets and Desserts	Frozen Yogurt	½ cup	1
Sweets and Desserts	Vanilla Wafers	5 wafers	1
Sweets and Desserts	Fruit Cobbler	½ cup	3
Sweets and Desserts	Pie (8 inches)	1/8	3
Fruit and Juices	Orange/Apple Juice	½ cup	1
Fruit and Juices	Apple/Banana	1 small	1
Fruit and Juices	Peach	1 medium	1
Fruit and Juices	Pear, large	½	1
Fruit and Juices	Blueberries/Blackberries	¾ cup	1
Fruit and Juices	Raspberries	1 cup	1
Fruit and Juices	Strawberries	1.25 cup	1
Fruit and Juices	Grapefruit, Large	1/2	1
Fruit and Juices	Cantaloupe, medium	1/3 melon	1
Fruit and Juices	Honeydew cubes	1 cup	1
Fruit and Juices	Grape/Prune Juice	1/3 cup	1
Milk and Yogurt	Cow or Soy milk	1 cup	1
Milk and Yogurt	Yogurt, Plain	1 cup	1
Milk and Yogurt	Yogurt with Fruit	½ cup	1

* 1 carbohydrate choice is approximately equal to 15 g of carbohydrates.

**Table 2:** Characteristics of participants in the Cooking Well with Diabetes Program (*n* = 1574).

Participant Characteristics		*n* (%)
Age (years)	18–24	31 (2.5)
	25–34	63 (4.0)
	35–44	133 (8.4)
	45–54	211 (13.4)
	55–64	390 (24.8)
	≥65	706 (44.9)
	n/a	40 (2.5)
Gender	Male	283 (18.0)
	Female	1195 (75.9)
	n/a	96 (6.1)
Race/Ethnicity	Hispanic	706 (44.9)
	White	550 (34.9)
	African American	227 (14.4)
	American Indian/Alaska Native	19 (1.2)
	Other *	35 (2.3)
	n/a	37 (2.4)
Have diagnosed diabetes	Yes	755 (48.0)
	No	737 (46.8)
	n/a	82 (5.2)
Prepare meals for someone with diabetes	Yes	517 (32.8)
	No	943 (59.9)
	n/a	114 (7.2)
Primary food shopper for the household	Yes	1342 (85.3)
	No	144 (9.1)
	n/a	88 (5.6)

n/a = not available due to missing data, * Includes Asian, multiracial, Native Hawaiian or Pacific Islander, or other.

**Table 3:** Changes in participants’ self-reported health, physical activity, and nutritional behaviors before and after participation in the Cooking Well with Diabetes Program (*n* = 1574).

Participant Self-Reported Health Behaviors		Pre *n* (%)	Post *n* (%)	*p*
Perception of general health	Excellent/very good/good	1025 (65.1)	1158 (73.6)	<0.001
	Fair/poor	549 (34.9)	416 (26.4)	
Engage in moderate/vigorous intensity physical activity in the past month	Yes	799 (50.8)	1040 (66.1)	<0.001
	No	643 (40.9)	414 (26.3)	
Portion of dinner plate filled with fruits and vegetables	More than 1/2	539 (34.2)	740 (47.0)	<0.001
	Less than 1/2	964 (61.2)	775 (49.2)	
Fruit consumption	≥2 times a day	548 (34.8)	772 (49.0)	<0.001
	<2 times a day	974 (61.9)	758 (48.2)	
Vegetable consumption	≥2 times a day	739 (47.0)	979 (62.2)	<0.001
	<2 times a day	759 (48.2)	555 (35.3)	
Regular soda consumption	Never	752 (47.8)	798 (50.7)	<0.001
	1–6 times a week	488 (31.0)	463 (29.4)	
	≥1 time a day	308 (19.6)	245 (15.6)	
Sweetened beverage consumption	Never	655 (41.6)	739 (47.0)	<0.001
	1–6 times a week	469 (29.8)	468 (29.7)	
	≥1 time a day	346 (22.0)	296 (18.8)	

**Table 4:** Changes in participants’ frequency of healthy cooking methods before and after participating in the Cooking Well with Diabetes Program (*n* = 1574).

Frequency of Food Consumption and Food Practices (Always/Mostly)	Pre *n* (%)	Post *n* (%)	*p*
Add salt to food at the table	268 (17.0)	245 (15.6)	<0.001
Replace salt with herbs and spices	526 (33.4)	734 (46.6)	<0.001
Replace shortening/lard with vegetable oil	1076 (68.4)	1268 (80.6)	<0.001
Modify recipes to lower sugar or fat	513 (32.6)	684 (43.5)	<0.001
Modify recipes to increase fiber	338 (21.5)	521 (33.1)	<0.001
Use healthy cooking methods instead of frying	869 (55.2)	1140 (72.4)	<0.001
Add extra vegetables to casseroles and soups	581 (36.9)	837 (53.2)	<0.001
Plan meals using the MyPlate method	245 (15.6)	507 (32.2)	<0.001
Check nutrition facts label on food packages	544 (34.6)	847 (53.8)	<0.001
Prepare meals and snacks at home	994 (63.2)	1148 (72.9)	<0.001

## Data Availability

The datasets presented in this article are not readily available because it is managed by the Office of Data and Accountability at Texas A&M AgriLife Extension Service. The data presented in this study are available on request from the corresponding author.
